# Successful Bentall Surgery in a Moyamoya Disease Patient with Intraoperative Brain Protection via IABP-Induced Pulsatile Flow and CO_2_ Management during Cardiopulmonary Bypass

**DOI:** 10.70352/scrj.cr.25-0664

**Published:** 2025-12-09

**Authors:** Kazuma Handa, Toshihiro Ohata, Tomohiko Sakamoto, Yukiko Arai, Tomoko Fujimoto, Akihiko Maeda, Teruya Nakamura, Toru Kuratani

**Affiliations:** 1 Department of Cardiovascular Surgery, Sakurabashi Watanabe Advanced Healthcare Hospital, Osaka, Osaka, Japan; 2 Department of Cardiovascular Surgery, The University of Osaka Graduate School of Medicine, Suita, Osaka, Japan; 3 Department of Anesthesiology, Sakurabashi Watanabe Advanced Healthcare Hospital, Osaka, Osaka, Japan

**Keywords:** moyamoya disease, intra-aortic balloon pumping, cardiopulmonary bypass, PexCO_2_, brain protection

## Abstract

**INTRODUCTION:**

Moyamoya disease is an occlusive cerebrovascular disorder in which maintaining cerebral blood flow during cardiac surgery is challenging. No reports have described Bentall surgery in such patients. We present a successful case using an integrated brain protection strategy combining intra-aortic balloon pumping (IABP)-induced pulsatile flow, carbon dioxide management, and moderate hypothermia.

**CASE PRESENTATION:**

A 51-year-old male with symptomatic moyamoya disease was referred for severe aortic regurgitation due to left coronary cusp leaflet shortening and a sinus of Valsalva aneurysm (50.7 mm) with cardiac dysfunction and left ventricular enlargement. Selective carotid angiography revealed poor visualization of the circle of Willis, including the anterior and middle cerebral arteries, along with the presence of moyamoya vessels. Oxygen PET imaging revealed Powers Stage II, indicating a high risk of cerebral infarction and intracranial hemorrhage. To maintain cerebral blood flow during cardiopulmonary bypass (CPB), intra-aortic balloon pumping was used to induce pulsatile flow, with moderate hypothermia for brain protection. Continuous monitoring of the partial pressure of carbon dioxide in the oxygenator exhaust gas allowed timely management of the arterial carbon dioxide during CPB, and cerebral oxygen saturation was used to optimize cerebral blood flow. Under this comprehensive brain flow management strategy, Bentall surgery was successfully performed. Postoperative MRI revealed no evidence of brain hemorrhage or infarction, and the patient was discharged without any neurological symptoms.

**CONCLUSIONS:**

This case demonstrates that IABP-induced pulsatile flow, PexCO_2_-guided CO_2_ management, and moderate hypothermia can effectively preserve cerebral circulation during CPB. This integrated approach enabled the safe and successful completion of Bentall surgery in a patient with moyamoya disease.

## INTRODUCTION

Moyamoya disease is a cerebrovascular disorder characterized by progressive stenosis of the terminal portions of the internal carotid arteries (ICAs), resulting in an increased risk of cerebral infarction due to reduced cerebral blood flow, as well as hemorrhage caused by the rupture of fragile collateral vessels.^1)^ The significant changes in cerebral blood flow, resulting from the continuous flow generated by standard cardiopulmonary bypass (CPB), pose a major concern. Pulsatile flow during CPB generates greater hemodynamic energy than non-pulsatile flow, reduces cerebrovascular resistance, and helps maintain adequate cerebral blood flow.^2)^ We present the case of a successful Bentall procedure in a patient with moyamoya disease, in which an integrated brain protection strategy was implemented. This strategy involved continuous monitoring of cerebral oxygen saturation (rSO_2_) and the partial pressure of carbon dioxide in the oxygenator exhaust gas (PexCO_2_), along with the use of intra-aortic balloon pumping (IABP) to induce pulsatile flow and moderate hypothermia to decrease brain metabolism during CPB.

## CASE PRESENTATION

**Fig. 1:**
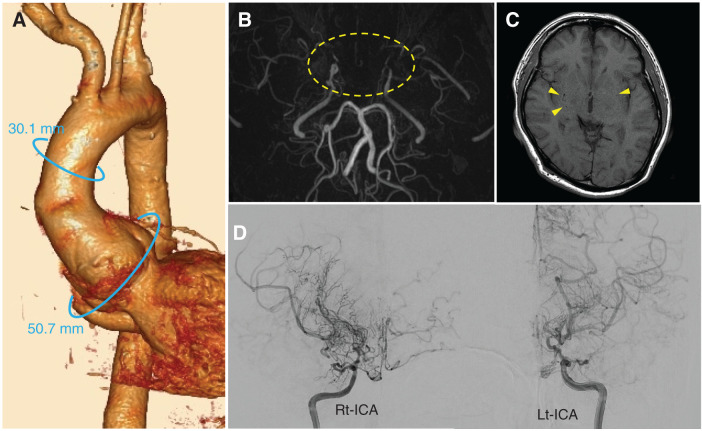
Preoperative findings. (**A**) Preoperative cardiac CT showed a sinus of Valsalva aneurysm, with no enlargement of the ascending aorta. (**B**) MR angiography revealed occlusion of bilateral ICAs, with no visualization of the circle of Willis, ACA, or MCA (dotted area). (**C**) T1-weighted images revealed small flow voids in the basal ganglia, indicating abnormal vascular networks (arrowhead). (**D**) Selective internal carotid angiography showed poor visualization of the circle of Willis, including ACA and MCA, and the presence of moyamoya vessels. ACA, anterior cerebral artery; ICA, internal carotid artery; Lt, left; MCA, middle cerebral artery; MR, magnetic resonance; Rt, right

**Fig. 2:**
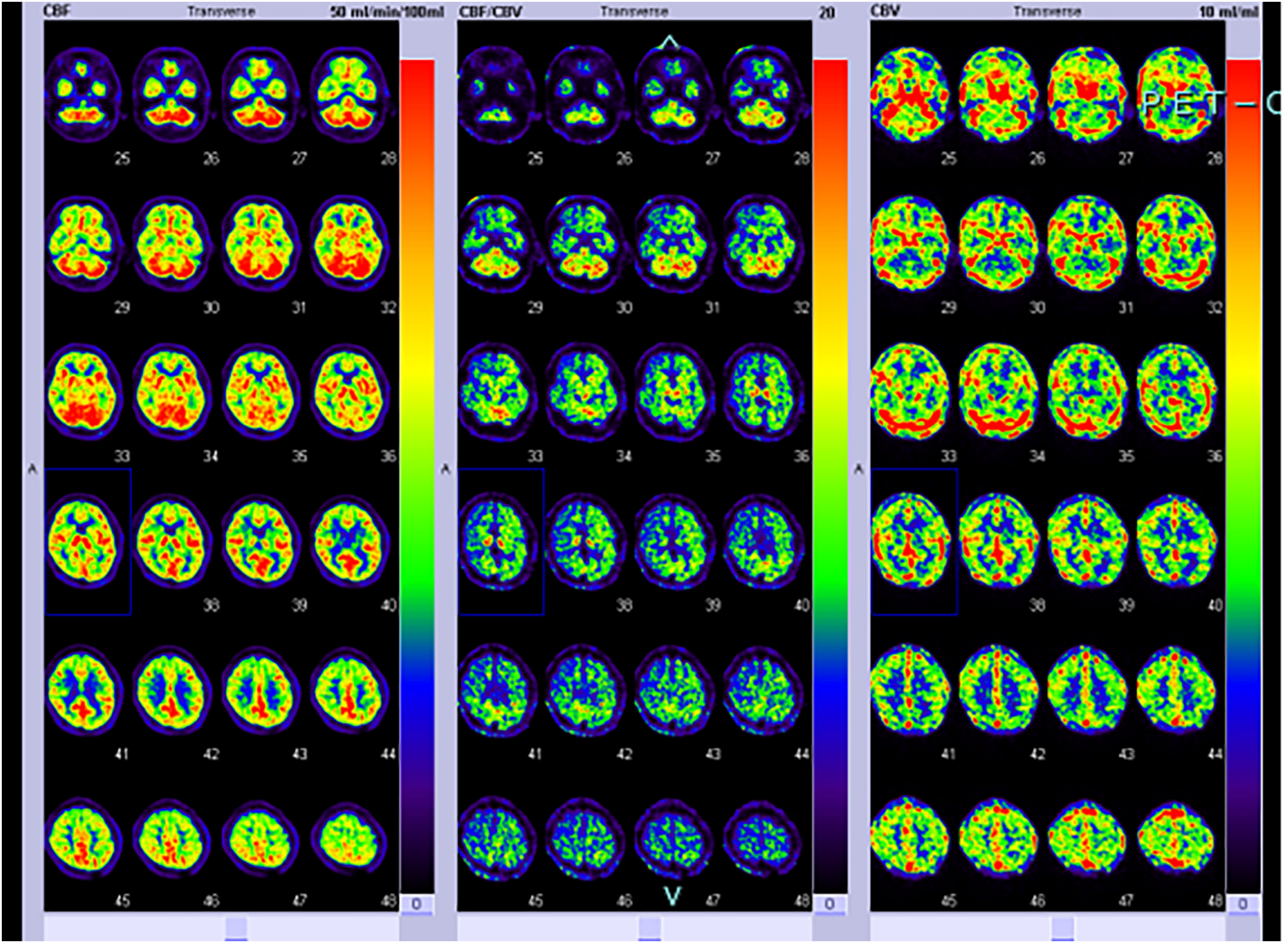
Preoperative oxygen PET imaging. CBF (left), CBF/CBV (center), CBV (right). Oxygen PET imaging revealed an increase in cerebral blood volume, a decrease in perfusion pressure, and a relative increase in oxygen extraction fraction, consistent with Powers Stage II, while no significant decrease in cerebral blood flow was observed. And the neurosurgeons recommended proceeding with aortic root replacement without preventive cerebral artery bypass surgery. CBF, cerebral blood flow; CBV, cerebral blood volume

A 51-year-old male was referred to our hospital due to cardiomegaly detected on a chest X-ray. His medical history included moyamoya disease, with transient ischemic attacks manifesting as upper limb weakness on exertion, occurring approximately once a year. Echocardiography revealed severe aortic regurgitation due to shortening of the left coronary cusp leaflet, along with cardiac dysfunction (ejection fraction, 34%) and diffuse left ventricular hypokinesis, as well as left ventricular enlargement (left ventricular diameter in diastole, 71 mm). Cardiac CT revealed a sinus of Valsalva aneurysm (50.7 mm), while the ascending aorta diameter remained normal (30.1 mm) (**Fig. 1A**). Head CT showed no hemorrhagic lesions, and magnetic resonance (MR) angiography demonstrated occlusion of the bilateral ICA, with no visualization of the circle of Willis, anterior cerebral artery (ACA), or middle cerebral artery (MCA) (**Fig. 1B**). T1-weighted MRI identified small flow voids in the basal ganglia, suggesting abnormal vascular networks (**Fig. 1C**). Selective carotid angiography revealed poor visualization of the circle of Willis, including the ACA and MCA, and showed the presence of moyamoya vessels (**Fig. 1D**). Oxygen PET imaging revealed an increase in cerebral blood volume, a decrease in perfusion pressure, and a relative increase in oxygen extraction fraction, consistent with Powers Stage II (**Fig. 2**). No significant decrease in cerebral blood flow was observed, and the neurosurgeons recommended proceeding with aortic root replacement without preventive cerebral artery bypass surgery. To optimize cerebral blood flow and prevent cerebrovascular spasm during CPB, an IABP was used to generate pulsatile flow, and both rSO_2_ and PexCO_2_ were continuously monitored to assess cerebral blood flow and arterial CO_2_ levels.

**Fig. 3:**
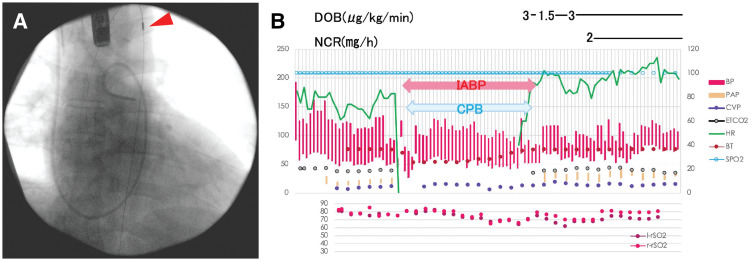
Intraoperative findings. (**A**) Insertion of the IABP via the femoral artery under fluoroscopic guidance at the time of anesthesia induction (arrowhead). (**B**) Serial changes in various parameters throughout the procedure. BP, blood pressure; BT, body temperature; CPB, cardiopulmonary bypass; CVP, central venous pressure; DOB, dobutamine; ETCO_2_, end-tidal carbon dioxide; HR, heart rate; IABP, intra-aortic balloon pumping; l-rSO_2_, left regional tissue oxygen saturation; NCR, nicardipine; PAP, pulmonary artery pressure; r-rSO_2_, right regional tissue oxygen saturation; SPO_2_, percutaneous oxygen saturation

The IABP was inserted at the time of anesthesia induction (**Fig. 3A**). A median sternotomy was performed, followed by ascending aorta and bicaval cannulation. The IABP was initiated at 60 beats/min to continuously generate pulse pressure upon initiation of CPB, maintaining an average blood pressure of 70–80 mmHg. Moderate hypothermia (25°C) was induced to prepare for potential hypotension. PexCO_2_ was strictly maintained at 40 mmHg, and rSO_2_ was kept above 60% (**Fig. 3B**). The baseline rSO_2_ was approximately 70%, with a minimum value of 62%. No significant fluctuations were observed during major operative phases, including CPB initiation and weaning, and rSO_2_ remained stable at around 70% throughout the procedure (**Fig. 3B**). The Bentall procedure was performed using a composite graft consisting of an INSPIRIS RESILIA aortic bioprosthesis (Edwards Lifesciences, Irvine, CA, USA) 23 mm and a J-graft Valsalva (Japan Lifeline, Tokyo, Japan) 26 mm under cross-clamping, with the Carrel patch technique for coronary artery reconstruction.

**Fig. 4:**
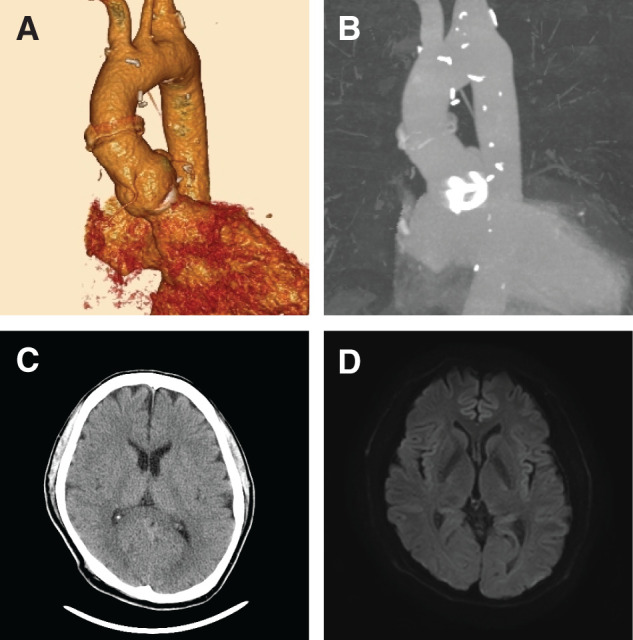
Postoperative imaging findings. (**A**, **B**) Cardiac CT following the Bentall procedure (**A**) and MIP images (**B**). (**C**) Head CT showing no hemorrhagic lesions. (**D**) Postoperative MRI diffusion-weighted images showing no evidence of infarction. MIP, maximum intensity projection

The aortic cross-clamp time and CPB time were 156 and 170 minutes, respectively. The IABP was weaned off and removed after confirming stable hemodynamics following CPB weaning. There were no postoperative neurological complications, and the patient was extubated on POD 2. Cardiac CT revealed no significant findings (**Fig. 4A** and **4B**), and postoperative head CT and MRI showed no signs of bleeding or infarction (**Fig. 4C** and **4D**). The patient was discharged without complications on POD 18, and no neurological symptoms were observed after discharge.

## DISCUSSION

Cardiac surgery in patients with moyamoya disease is particularly challenging due to the significant risks of cerebral infarction from intraoperative hypotension and the continuous flow of CPB, as well as the risk of bleeding from elevated blood pressure and systemic heparinization. While previous reports have addressed off-pump coronary artery bypass grafting,^3)^ mitral valve repair,^4)^ and ascending aortic replacement^5)^ in moyamoya disease patients, no reports have yet described aortic root replacement (**Table 1**). This case represents a rare and successful instance of Bentall surgery in a moyamoya disease patient, where comprehensive cerebral protection strategies—such as IABP, moderate hypothermia, and CO_2_ management—proved effective during the aortic root replacement.

**Table 1:** Previous reports of cardiac surgery in patients with moyamoya disease

Author	Age ⋅ sex	Procedure	CPB time	IABP use	Hypothermia	BP during CPB	CO_2_ management during CPB	rSO_2_ range
Komiyama, et al.^3)^	56 ∙ F	MIDCAB	N/A	N/A	N/A	Systolic ≥ 100 mmHg	N/A	N/A
Okamoto, et al.^4)^	51 ∙ F	MV repair	158 min	Yes	Mild (32–34°C)	Mean ≥ 70 mmHg	PaCO_2_ 40–45 mmHg	35%–70%
Kuwajima, et al.^5)^	9 ∙ F	GR of Asc.Ao	546 min (DHCA 45 min)	No	Deep (18°C)	Mean 40–65 mmHg	PaCO_2_ 40–50 mmHg	50%–80%
Current case	51 ∙ M	Bentall	170 min	Yes	Moderate (25°C)	Mean 70–80 mmHg	PexCO_2_ 40 mmHg	60%–80%

Asc.Ao, ascending aorta; BP, blood pressure; CO_2_, carbon dioxide; CPB, cardiopulmonary bypass; DHCA, deep hypothermia circulatory arrest; GR, graft replacement; IABP, intra-aortic balloon pumping; MIDCAB, minimally invasive direct coronary artery bypass; MV, mitral valve; PexCO_2_, partial pressure of carbon dioxide in the oxygenator exhaust gas; rSO_2_, cerebral oxygen saturation

Given the patient’s young age, valve-sparing aortic root replacement was initially considered. However, the etiology of the aortic regurgitation was attributed to shortening of the left coronary cusp leaflet, which would have required extended time for reconstruction. Since prolonged CPB time increases the risk of cerebral complications, minimizing cross-clamp time was prioritized, leading to the decision to perform a Bentall procedure with a prosthetic valve. Furthermore, while a mechanical valve would typically be chosen for a patient of this age, a bioprosthesis was selected to mitigate the risk of long-term cerebral complications, as it does not require anticoagulation therapy. On the other hand, the freedom from structural valve deterioration in patients in their 50s undergoing bioprosthetic valve implantation has been reported to be approximately 79% at 10 years and 57% at 15 years.^6)^ Therefore, long-term structural deterioration remains a significant concern in this patient, and careful longitudinal follow-up will be essential.

Regarding cerebral circulation management, pulsatile flow is more effective than continuous flow in maintaining cerebral blood flow during CPB.^2,7)^ The use of IABP alongside standard continuous-flow CPB to induced pulsatile flow has also been demonstrated to improve organ perfusion.^8)^ Therefore, we utilized IABP during this procedure. On the other hand, cardiac surgery carries the risk of hypotension due to bleeding, vasoplegia, and heart failure. To mitigate the potential for unexpected hypotension, moderate hypothermia (25°C) was induced to reduce cerebral oxygen consumption. Although deep hypothermia can provide greater tolerance to cerebral ischemia, it may also increase the risk of mortality due to coagulopathy and end-organ injury. Current recommendations for aortic arch surgery involving circulatory arrest suggest using moderate hypothermia at 24°C–28°C.^9)^ When prolonged cerebral ischemia is anticipated, selecting a temperature at the lower end of this range is considered reasonable. Based on these considerations, we selected a target temperature of 25°C.Additionally, a decrease in blood CO_2_ levels can cause cerebral artery spasm, leading to ischemia, making CO_2_ management particularly important in moyamoya disease. In addition to frequent arterial blood gas analyses, continuous monitoring of CO_2_ during CPB was achieved by measuring CO_2_ at the PexCO_2_, which correlates with PaCO_2_.^10)^ The mean difference between PexCO_2_ and PaCO_2_ was 2.3 mmHg, indicating that the 2 values were nearly identical.^10)^ Therefore, maintaining PexCO_2_ at 40 mmHg can be regarded as essentially equivalent to controlling PaCO_2_ at 40 mmHg. Continuous quantitative assessment of cerebral blood flow was performed using rSO_2_. These comprehensive cerebral blood flow management strategies during CPB in moyamoya disease patients have the potential to be applied not only to aortic root replacement, as demonstrated in this case, but also to most cardiac surgeries.

## CONCLUSIONS

In patients with moyamoya disease, a meticulous and comprehensive cerebral protection strategy was employed, including the creation of pulsatile flow with IABP during CPB, moderate hypothermia to prepare for unexpected hypotension, strict management of arterial CO_2_ levels through continuous monitoring of PexCO_2_ to prevent arterial spasm, and continuous cerebral blood flow assessment using rSO_2_. This strategy effectively managed cerebral circulation, contributing to the successful and safe performance of the Bentall procedure.

## DECLARATIONS

### Availability of data and materials

The authors declare that all data in this article are available within the article.

## References

[1] Scott RM, Smith ER. Moyamoya disease and moyamoya syndrome. N Engl J Med 2009; 360: 1226–37. 19297575 10.1056/NEJMra0804622

[2] Ündar A, Masai T, Beyer EA, et al. Pediatric physiologic pulsatile pump enhances cerebral and renal blood flow during and after cardiopulmonary bypass. Artif Organs 2002; 26: 919–23. 12406143 10.1046/j.1525-1594.2002.07127.x

[3] Komiyama M, Ishikawa T, Takanashi S, et al. Minimal invasive direct coronary artery bypass in moyamoya disease. Interact Cardiovasc Thorac Surg 2003; 2: 65–7. 17669990 10.1016/S1569-9293(02)00100-7

[4] Okamoto K, Arai S, Hirabayashi N, et al. Cerebral protection during mitral valve repair in a patient with moyamoya syndrome. Ann Thorac Surg 2015; 99: 2208–10. 26046880 10.1016/j.athoracsur.2014.08.049

[5] Kuwajima K, Yoshitani K, Kato S, et al. Deep hypothermic circulatory arrest for hemiarch replacement in a pediatric patient with moyamoya disease. J Anesth 2014; 28: 613–7. 24398624 10.1007/s00540-013-1782-6

[6] Forcillo J, El Hamamsy I, Stevens LM, et al. The perimount valve in the aortic position: twenty-year experience with patients under 60 years old. Ann Thorac Surg 2014; 97: 1526–32. 24681031 10.1016/j.athoracsur.2014.02.019

[7] Su XW, Guan Y, Barnes M, et al. Improved cerebral oxygen saturation and blood flow pulsatility with pulsatile perfusion during pediatric cardiopulmonary bypass. Pediatr Res 2011; 70: 181–5. 21544006 10.1203/PDR.0b013e3182226b75

[8] Serraino GF, Marsico R, Musolino G, et al. Pulsatile cardiopulmonary bypass with intra-aortic balloon pump improves organ function and reduces endothelial activation. Circ J 2012; 76: 1121–9. 22447003 10.1253/circj.cj-11-1027

[9] Zhang K, Zhou C, Gao S, et al. The optimal degree of core temperature for hypothermic circulatory arrest in complex aortic arch surgery: results from 1310 patients. Eur J Cardiothorac Surg 2024; 66: ezae311. 39137134 10.1093/ejcts/ezae311

[10] Baraka A, El-Khatib M, Muallem E, et al. Oxygenator exhaust capnography for prediction of arterial carbon dioxide tension during hypothermic cardiopulmonary bypass. J Extra Corpor Technol 2005; 37: 192–5. 16117458 PMC4682535

